# Association of hyperuricemia combined with sarcopenia on ASCVD risk

**DOI:** 10.1186/s12872-023-03336-2

**Published:** 2023-06-27

**Authors:** Guqiao Nie, Jingjing Wan, Lei Jiang, Meng Zhang, Fengqin Yan, Wen Peng

**Affiliations:** Department of General Practice, Union Hospital, Tongji Medical College, Huazhong University of Science and Technology, Jie Fang avenue, WuHan, Hubei 1227 China

**Keywords:** Atherosclerotic cardiovascular disease, Sarcopenia, Hyperuricemia, Insulin resistance

## Abstract

**Background:**

Hyperuricemia and sarcopenia are both strongly linked to an increased risk of atherosclerotic cardiovascular disease (ASCVD), and this study was designed to look into the interactive effects of hyperuricemia on ASCVD risk.

**Methods:**

This study collected information from patients (*N* = 2647) who underwent health check-ups at the Health Care Building of Wuhan Union Hospital between January 2019 and December 2020. Skeletal muscle mass was measured using bioelectrical impedance methods. The Asian Working Group on Sarcopenia diagnostic criteria were used to classify patients with sarcopenia. ASCVD risk was calculated using the Framingham Heart Study, and ASCVD risk ≥ 20% was considered high risk ASCVD. IBM SPSS 25.0 and GraphPad prism 8.0 software were used for data analysis and graphing.

**Results:**

The prevalence of hyperuricemia and sarcopenia was 23.57% and 15.34%, respectively. The occurrence of cardiovascular risk factors such as obesity, hypertension, diabetes mellitus, chronic kidney disease, and low HDL-Cemia was significantly higher in subjects with hyperuricemia combined with sarcopenia (OR = 1.734, 3.064, 1.61, 8.77 and 1.691 respectively, *p *< 0.05); Hyperuricemia and high-risk ASCVD were independently associated (OR = 1.355, 95% CI = 1.000–1.838, *p* = 0.04). Although there was no significant association between sarcopenia and high-risk ASCVD after controlling for confounders (OR = 1.274, 95% CI = 0.828–1.959, *p* = 0.271), sarcopenia combined with hyperuricemia significantly increased high-risk ASCVD (OR = 3.229, 95% CI 1.544–6.751, *p* = 0.002).

**Conclusion:**

Hyperuricemia is independently associated with high-risk ASCVD; Sarcopenia and high-risk ASCVD did not show an independent relationship, but there was a synergistic effect of the two on ASCVD risk, which may imply that managing both hyperuricemia and sarcopenia may have a greater cardiovascular benefit.

**Supplementary Information:**

The online version contains supplementary material available at 10.1186/s12872-023-03336-2

## Background

Hyperuricemia, a chronic metabolic disease, plays a critical role in the development of gout and has become a global health problem, with hyperuricemia prevalence increasing in China [1]. There is strong epidemiological evidence linking hyperuricemia to cardiovascular events and all-cause mortality from cardiovascular events [2–5]. Management guidelines from the European Society of Cardiology (ESC) and the European Society of Hypertension (ESH) have officially recognized uric acid as a risk factor for cardiovascular disease [6]. Furthermore, it has been demonstrated that altered insulin resistance in hyperuricemia patients contributes to the development of atherosclerotic cardiovascular disease (ASCVD) [7, 8].

Sarcopenia is a progressive and systemic skeletal muscle disease that can lead to adverse outcomes such as falls, disability, frailty in old age, and hospital mortality [9, 10]. The prevalence of sarcopenia is increasing in an aging population [11, 12]. It has been shown that sarcopenia is strongly associated with the risk of cardiovascular events in patients with type 2 diabetes and chronic kidney disease [13–15]. Heart failure and sarcopenia are thought to be causal and interact in studies [16]. Changes in insulin sensitivity, inflammatory factors, and oxidative stress in sarcopenic patients may play a role in the development of cardiovascular disease [17–19].

The relationship between sarcopenia and hyperuricemia has also received attention in recent years. Higher uric acid levels were found to be associated with sarcopenia in a study of a western Chinese population; high uric acid levels were also associated with reduced skeletal muscle mass in men with diabetes [20, 21]. However, the interaction of hyperuricemia and sarcopenia on CVD risk has not been studied. We investigated the interaction between hyperuricemia and sarcopenia on the risk of CVD in a population independently of other metabolic factors (smoking, alcohol consumption, obesity, hypertension, type II diabetes mellitus(T2DM), chronic kidney disease (CKD), and dyslipidemia).

### Population and Methods

#### Study population

A total of 3011 patients who underwent health screening at the Health Care Building of the Wuhan Union Hospital, Wuhan, China, between January 2019 and December 2020.

Inclusion criteria: age ≥ 50 years; Asian population. The loss of skeletal muscle mass in the human body begins gradually around the age of 50 [9, 22], so the age of our study population is divided into years old and over 50.

Exclusion criteria: insufficient information on clinical and laboratory tests; insufficient data to calculate the risk of atherosclerotic cardiovascular disease (ASCVD); being in acute infection; active rheumatic immune disease; on hormone therapy; in hemodialysis for renal failure; diagnosis of malignancy.

The above screening resulted in a total of 2647 inpatients enrolled in the study; the study fully complied with the Declaration of Helsinki and was approved by the ethics committee of Huazhong University of Science and Technology (2022-S155).

#### Definition of sarcopenia and hyperuricemia

Skeletal muscle mass and other body components were measured using body composition analysis instruments (Tsinghua Tongfang, BCA-2A, China). Due to limited data, we only have data on skeletal muscle mass. Definition of sarcopenia using the threshold values of the skeletal muscle mass index of the Asian Working Group on Sarcopenia [10]. The skeletal muscle mass index < 7.0 kg/m^2^ in men, and 5.7 kg/m^2^ in women is classified as sarcopenia. Lee, Han et al. conducted sarcopenia-related research based on skeletal muscle mass division sarcopenia [13, 23]. Hyperuricemia was defined according to the Chinese Medical Association Endocrine Branch for diagnosis, that is, fasting serum uric acid levels greater than 420 μmol/L measured twice on non-same day [24].

#### ASCVD risk calculation

ASCVD risk was obtained according to the Framingham Heart Study (FHS)10-year ASCVD risk calculation formula by judging age, blood lipids (high density, total cholesterol), systolic blood pressure (hypertension medication status), smoking history and diabetes history, and finally the ASCVD risk score was obtained to calculate the corresponding risk proportion, greater than or equal to 20% we called high-risk ASCVD [25].

#### Clinical parameters and biochemical analysis

Patients who underwent health screening provided basic information such as their age, gender, smoking history, alcohol consumption history, previous illnesses, and medication use (statins, hypertension medications, and use of medications for diabetes treatment). Patient self-report classified smoking status as non-smoker or smoker. Following an overnight fast of at least 8 h, blood samples were collected from each subject and sent to the various departments of the hospital's laboratory for testing within 2 h. Biochemical tests collected include white blood cell(WBC),

Hemoglobin(Hb), (NE), lymphocyte (LY), alanine aminotransferase (ALT), aspartate aminotransferase (AST), albumin (ALB), creatinine (Cr), uric acid, fasting blood sugar (FBG), total cholesterol (TC), low-density lipoprotein (LDL-C), triglycerides (TG) and high-density lipoprotein (HDL-C). The NLR ratio is the neutrophil count to lymphocyte ratio. The TG/HDL ratio is the triglyceride to HDL cholesterol ratio. Glomerular filtration rate was determined using the MDRD equation eGFR (mL/min/1.73 m^2^) = 175 × Cr (mg/ dL) ^−1.154^ × Age(year) ^−0.203^ × 0.742(female) [26]. CKD defined as eGFR < 60 mL/min/1.73 m^2^.

### Statistical analysis

The study population was divided into four groups based on hyperuricemia and sarcopenia status, and one-way ANOVA and nonparametric tests were used to compare normally and non-normally distributed continuous variables, chi-square tests were used for categorical variables, and post hoc analyses were performed using the Bonferroni method and the Kruskal–Wallis H test. The results of a logistic regression analysis were presented as forest plots to help identify risk factors for high-risk ASCVD. With confounders controlled for, multiple logistic regression analysis was used to determine the independent association between hyperuricemia/sarcopenia and high ASCVD risk.

Nonparametric tests were used because the levels of ALT, AST, ALB, TG/HDL ratio, and glomerular filtration rate were not normally distributed. The continuous and categorical variables were expressed as mean standard deviation (SD) and percentage (%), respectively. The median was used to represent continuous variables that did not have a normal distribution (first quartile, third quartile). *P* values less than 0.05 were considered statistically significant.

## Result

### Baseline characteristics of the study population

**Fig. 1:**
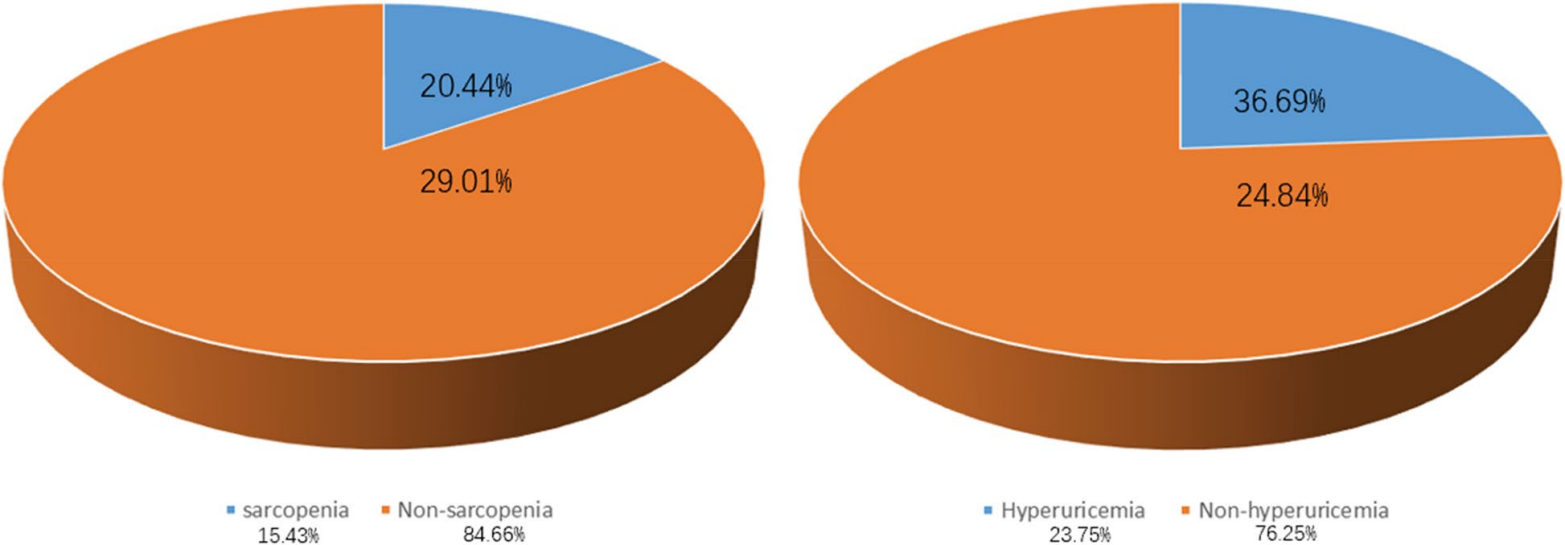
Distribution of high-risk ASCVD in the sarcopenic and hyperuricemic populations ASCVD(%)

There were 2647 subjects (875 women and 1772 men) in the study. The prevalence of hyperuricemia and sarccopenia was 23.57% (624 of 2647) and 15.34% (406 of 2647), respectively (Fig. 1). The proportion of high-risk ASCVD was 36.69% in hyperuricemic population. The proportion of high-risk ASCVD was 20.44% in sarcopenic population (Fig. 1). All subjects were divided into four categories according to hyperuricemia and sarcopenia status (non-hyperuricemia and non-sarcopenia group, hyperuricemia and non-sarcopenia group, sarcopenia and non-hyperuricemia group, and sarcopenia and hyperuricemia group). Compared to the group without diagnosis of hyperuricemia and sarcopenia (Table 1), subjects with both hyperuricemia and sarcopenia had significantly increased age, WBC level, creatinine, and TG/HDL ratio (*p* < 0.001), while body mass index(BMI)、Hb level、ALB level、eGFR level were decreased (*p *< 0.05). The proportion of hypertension, diabetes, smokers, alcohol drinkers, and obese people was significantly higher in the hyperuricemia and sarcopenia groups (all *p* < 0.05). Skeletal muscle mass in the group (sarcopenia combined with hyperuricemia) is significantly lower than in the group that only diagnoses hyperuricemia (Table 1).

**Table 1:** Baseline characteristics of the study population

**Characteristics**	**Sarcopenia (-) hyperuricemia (-)**	**Sarcopenia ( +) hyperuricemia (-)**	**Sarcopenia (-) hyperuricemia ( +)**	**Sarcopenia ( +) hyperuricemia ( +)**	*p* value
Age	62.27 ± 9.96	65.91 ± 11.09^b,d^	62.39 ± 11.06^c,e^	70.98 ± 12.93^b,c,d^	< 0.001
Gender(male%)	991(61.55%)	258(62.47%)	427(83.73%)	96(84.21%)	*p* < 0.001
Smoking(Yes%)	419(27.05%)	101(25.44%)^d^	189(38.1%)^b,c^	33(29.2%)	*p* < 0.001
Alcohol(Yes%)	357(23.05%)	83(20.91%)^d,e^	192(38.71%)^b,c^	37(32.74%)^b,c^	*p* < 0.001
DM(Yes%)	440(27.33%)	137(33.17%)^e^	150(29.41%)^e^	43(37.71%)^b^	*p* = 0.019
Hypertension(Yes%)	845(54.72%)	255(64.39%)^b,e^	347(70.52%)^b^	88(78.57%) ^b,c^	*p* < 0.001
Obesity(Yes%)	176(10.93%)	57(13.80%)^b,e^	87(17.05%)^b^	20(17.54%)^b,c,d^	*p* = 0.001
Skletal muscle mass(kg)	22.15 ± 4.41	14.89 ± 3.55^b^^,^^d^	24.09 ± 3.98^b,c,e^	16.46 ± 3.72^b,d^	< 0.001
BMI(kg/m^2^	24.90 ± 2.85	19.74 ± 2.09^b,d^	25.73 ± 2.84^b,c,e^	19.81 ± 1.95^b,d^	< 0.001
WBC(G/L)	5.56 ± 1.37	5.12 ± 1.38	5.86 ± 1.4^b,c^	5.54 ± 1.37	< 0.001
Hb(g/dl)	136.24 ± 16.51	128.20 ± 19.19^b,d^	140.96 ± 17.69^b,c^	132.22 ± 18.81^d^	< 0.001
NE(G/L)	3.23 ± 1.10	3.01 ± 1.17^b,d^	3.42 ± 1.09^b,c^	3.32 ± 1.03	< 0.001
LY(G/L)	1.77 ± 0.53	1.62 ± 0.55^b,d^	1.84 ± 0.54^b,c^	1.71 ± 0.64	0.006
NRL	1.99 ± 1.01	2.13 ± 1.56	2.02 ± 0.97	2.23 ± 1.42	0.082
ALT(U/L)	20(14, 28)	15(11, 21)^b,d,e^	23(16, 31.25)^b,c,e^	15.5(11, 23.25)^b,c,d^	< 0.001
AST(U/L)	21(17, 26)	19(16.5, 24)^b,d^	21(18, 27)^c^	20(17, 27)	< 0.001
ALB(g/L)	41.1(38.8, 43.6)	40.8(38.3, 43.85)^d,e^	41.9(39.6, 44.6)^c,e^	38.9(36.3, 41.275)^b,c,d^	< 0.001
eGFR (mL/min/1.73 m^2^)	92.88(80.81,105.02)	92.32(81.00,105.89)^d,e^	83.17(69.05,97.55)^b,c^	74(57.16,95.57)^b,c^	0.005
Cr(μmol/L)	71.34 ± 20.62	65.09 ± 15.76^d,e^	85.63 ± 27.51^b,c^	88.26 ± 31.27^b,c^	< 0.001
Uric acid(μmol/L)	319.36 ± 66.86	284.5869.09^b,d,e^	486.27 ± 58.11^b,c^	471.04 ± 46.42^b,c^	< 0.001
FBG(mmol/L)	5.01(4.6, 5.7)	4.8(4.4375, 5.3)^b,d^	5.15(4.66, 5.8)^c^	4.925(4.6, 5.775)	< 0.001
TC(mmol/L)	4.07 ± 1.49	4.56 ± 2.37^b^	4.33 ± 1.58^b^	4.28 ± 1.05	< 0.001
LDL-C(mmol/L)	2.44 ± 0.95	2.59 ± 0.96b	2.52 ± 0.99	2.48 ± 1.01	0.041
TG(mmol/L)	1.2(0.9, 1.73)	0.99(0.75, 1.32) ^b,d^	1.57(1.13, 2.4925)^b,c,e^	0.975(0.7075, 1.495)^d^	< 0.001
HDL-C(mmol/L)	1.16(0.96, 1.44)	1.32(1.075, 1.65)^b,d^	1.02(0.85, 1.24)^b,c,e^	1.155(0.9775, 1.4625)^d^	< 0.001
TG/HDL-C ratio	0.93(0.61, 1.54)	0.87(0.54, 1.59)^d,e^	1.56(0.93,2.52)^b,c,e^	1.31(0.83, 2.13)^b,c,d^	< 0.001
Statins	349(21.67%)	122(29.54%)^b^	121(23.72%)	34(29.82%)	*p* = 0.003
ARB	355(22.05%)	98(23.73%)^e^	156(30.58%)^b^	42(36.84%)^b,c^	*p* < 0.001
ACEI	74(4.59%)	26(6.29%)	30(5.88%)	8(7.02%)	*p* = 0.33
β-B	242(15.03%)	83(20.09%)^b,e^	79(15.49%)^e^	29(25.43%)^b,d^	*p* = 0.004
CCB	361(22.42%)	112(27.11%)^e^	148(29.01%)^b,e^	38(33.33%)^b^	*p* = 0.002
metformin	209(12.98%)	54(13.07%)	70(13.72%)	22(19.29%)	*p* = 0.291
Sulfonylureas	32(1.98%)	17(4.11%)^b,d^	7(1.37%)^c^	3(2.63%)	*p* = 0.03
glinides	2(0.1%)	1(0.2%)	1(0.1%)	0(0)	*p* = 0.909
thiazolidinediones	17(1.06%)	5(0.12%)	1(0.19%)	3(0.26%)	*p* = 0.081
α-glycosidase inhibitor	135(8.38%)	38(9.2%)	29(5.68%)	11(9.64%)	*p* = 0.156
DPP4	81(5.03%)	24(5.81%)	30(5.88%)	9(7.89%)	*p* = 0.539
GLP1	5(0.31%)	0(0)	3(0.58%)	0(0)	*p* = 0.394
SGLT2	45(2.79%)	10(2.42%)	10(1.96%)	3(2.63%)	*p* = 0.772
Insulin	78(4.84%)	17(4.11%)	18(3.53%)	4(3.51%)	*p* = 0.581

*Abbreviations*: *ASM* Appendicular skeletal muscle, *WBC* White blood cell, *Hb* Hemoglobin, *NE* Neutrophils, *LY* Lymphocyte, *ALT* Alanine aminotransferase, *AST* Aspartate aminotransferase, *ALB* Albumin, *Cr* Creatinine, *FBG* Fasting blood sugar, *TC* Total cholesterol, *LDL-C* Low-density lipoprotein, *TG* Triglycerides and *HDL-C* High-density lipoprotein, *BMI* Body mass index, *DM* Diabetes, *eGF* Estimated glomerular filtration rate, *ARB* Angiotensin II receptor blockers, *CCB* Calcium channel blockers, *ACEI* Angiotensin-converting enzyme inhibitors, *β-B* Beta-blockers

^a^Obesity was defined as BMI ≥ 25 kg/m2

^b^*P* < 0.0125 by post hoc analyses when compared without sarcopenia and hyperuricemia

^c^*P* < 0.0125 by post hoc analyses when compared with sarcopenia, without hyperuricemia

^d^*P* < 0.0125 by post hoc analyses when compared without sarcopenia, with hyperuricemia

^e^*P* < 0.0125 by post hoc analyses when compared with sarcopenia and hyperuricemia

### Logistic regression analysis of high-risk ASCVD risk factors

**Fig. 2:**
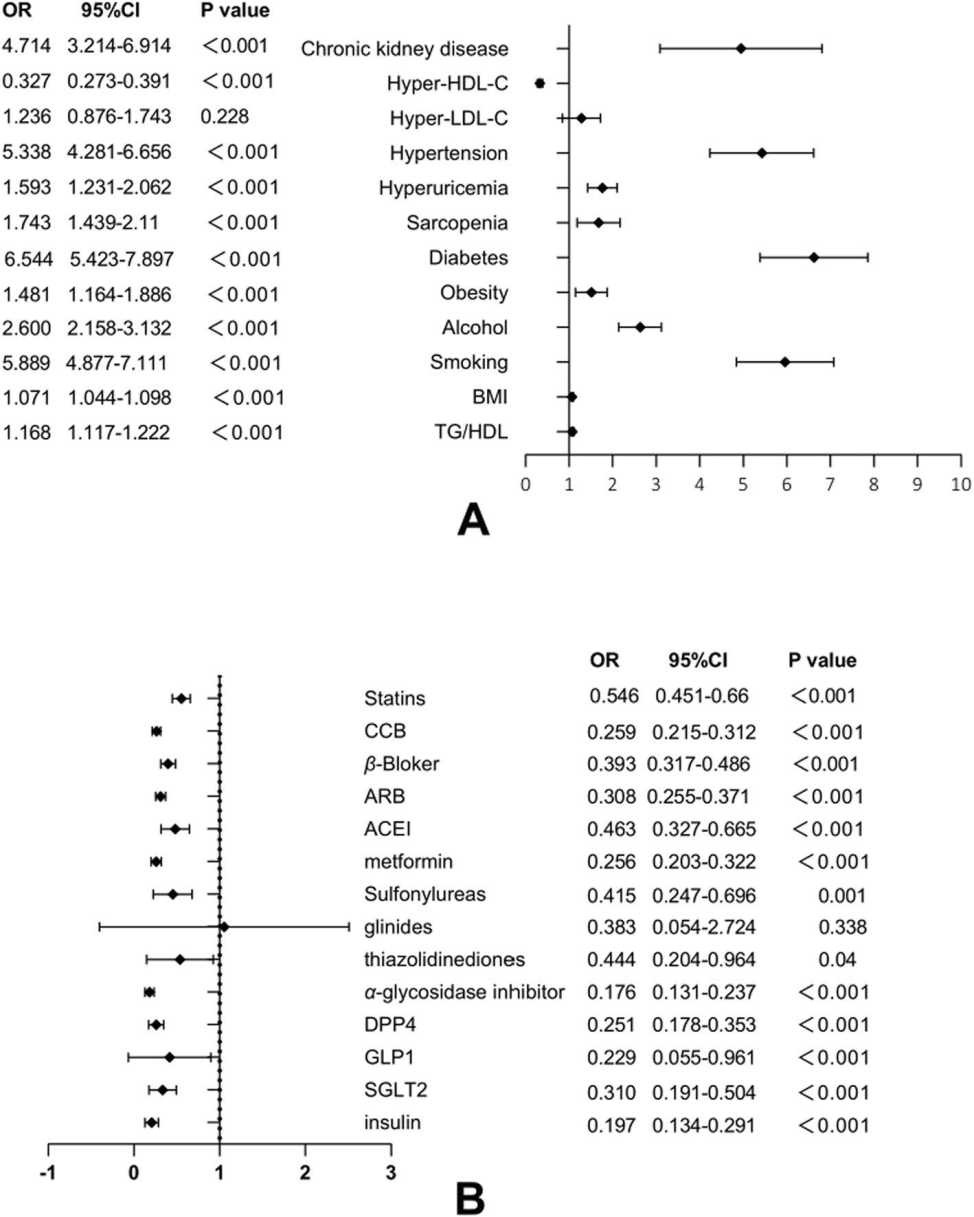
Logistic regression analysis of high-risk ASCVD risk factors

ASCVD is associated with many risk factors, and we conducted a logistic regression analysis between each variable and ASCVD risk separately, and the results were visualized in a forest plot (Fig. 2), with smoking (OR = 5.889, 95% CI = 4.887, 7.111), alcohol consumption (OR = 2.6, 95% CI = 2.158, 3, 132), obesity (OR = 1.481, 95% CI = 1.164, 1.886), hypertension (OR = 5.338, 95% CI = 4.281, 6.656), diabetes mellitus (OR = 6.544, 95% CI = 5.423, 7.897), hyperuricemia (OR = 1.743, 95% CI = 1.439, 2.11), sarcopenia (OR = 1.743, 95% CI = 1.439, 2.110), hyperHDL-Cemia (OR = 0.327, 95% CI = 0.273, 0.391), TG/HDL-C ratio (OR = 1.168, 95% CI = 1.117, 1.222) and high-risk ASCVD were positively associated, and the use of all antihypertensive medications, as well as the statin class, were protectively associated with high-risk ASCVD. The use of statins reduced high-risk ASCVD by 0.545-fold (OR = 0.545, 95% CI = 0.451–0.66), and the use of hypertensive drugs such as CCB and ACEI class also showed a protective trend (*p* ≤ 0.001 for all). Except for the use of glinide hypoglycemic agents, the use of other hypoglycemic agents showed protective relationship (*p* ≤ 0.001 for all).

### Association between cardiovascular risk factors and hyperuricemia/sarcopenia

Similarly, we divided the study population into four groups based on hyperuricemia and sarcopenia status and ran an association analysis to see if there was a link between grouping status and cardiometabolic risk factors. (Table 2). When not corrected for the effect of confounding factors, subjects with a concurrent diagnosis of hyperuricemia and sarcopenia had a relatively higher odds ratios of hypertension, diabetes, chronic kidney disease, obesity, low HDL cholesterolemia, and hypertriglyceridemia (OR 3.064, 1.61, 8.77, 1.734, 1.69, 2.116, all *p* < 0.05, respectively); Adjusted for age, sex, smoking, and alcohol consumption in the regression model, the relative risks of hypertension, chronic kidney disease, obesity, low HDL cholesterolemia, and hypertriglyceridemia,the odds ratios of occurrence was significantly increased (OR 1.862, 6.263, 1.649, 1.485 and 2.506, respectively, all *p* < 0.05).

**Table 2:** ORs and 95% CIs for traditional risk factor contributed by Hyperuricemia /Sarcopenia

	Model1				Model2			
Risk factors	**Sarcopenia (-) hyperuricemia (-)**	**Sarcopenia (-) hyperuricemia ( +)**	**Sarcopenia ( +) hyperuricemia (-)**	**Sarcopenia ( +) hyperuricemia ( +)**	**Sarcopenia (-) hyperuricemia (-)**	**Sarcopenia (-) hyperuricemia ( +)**	**Sarcopenia ( +) hyperuricemia (-)**	**Sarcopenia ( +) hyperuricemia ( +)**
Hypertension	1.0(ref)	OR = 1.927 1.562–2.378 *p* < 0.001	OR = 1.476 1.183–1.842 *p* = 0.001	OR = 3.064 1.957–4.797 *p* < 0.001	1.0(ref)	OR = 1.769 1.148–2.206 *p* < 0.05	OR = 1.048 0.827–1.328 *p* = 0.698	OR = 1.862 1.165–2.977 *p* < 0.05
Diabetes	1.0(ref)	OR = 1.108 0.889–1.38 *p* = 0.361	OR = 1.32 1.046–1.665 *p* = 0.019	OR = 1.61 1.086–2.389 *p* = 0.018	1.0(ref)	OR = 1.004 0.801–1.258 *p* = 0.973	OR = 1.125 0.884–1.433 *p* = 0.338	OR = 1.201 0.799–1.804 *p* = 0.379
CKD	1.0(ref)	OR = 3.842 2.687–5.439 *p* < 0.001	OR = 1.786 1.129–2.826 *p* = 0.013	OR = 8.77 5.388–14.273 *p* < 0.001	1.0(ref)	OR = 5.001 3.405–7.344 *p* < 0.05	OR = 1.078 0.672–1.728 *p* = 0.096	OR = 6.263 3.72–10.547 *p* < 0.001
Obesity	1.0(ref)	OR = 1.676 1.268–2.215 *p* < 0.001	OR = 1.305 0.947–1.797 *p* = 0.104	OR = 1.734 1.044–2.879 *p* = 0.034	1.0(ref)	OR = 1.499 1.127–1.992 *p* < 0.001	OR = 1.364 0.981–1.897 *p* = 0.065	OR = 1.649 1.01–2.476 *p* < 0.001
Hyper-LDL cholesterolemia	1.0(ref)	OR = 1.211 0.814–1.802 *p* = 0.344	OR = 0.897 0.557–1.446 *p* = 0.656	OR = 1.043 0.472–2.304 *p* = 0.917	1.0(ref)	OR = 1.294 0.858–1.95 *p* = 0.219	OR = 1.091 0.668–1.782 *p* = 0.728	OR = 1.438 0.637–3.248 *p* = 0.382
Hypo-HDL cholesterolemia	1.0(ref)	OR = 2.325 1.889–2.861 *p* < 0.001	OR = 1.013 0.792–1.297 *p* = 0.915	OR = 1.691 1.137–2.514 *p* = 0.009	1.0(ref)	OR = 2.011 1.624–2.49 *p* < 0.05	OR = 1.012 0.787–1.301 *p* = 0.927	OR = 1.485 1.291–2.225 *p* < 0.046
Hypertriglycerid-emia	1.0(ref)	OR = 2.043 1.219–3.39 *p* < 0.001	OR = 1.041 0.801–1.354 *p* = 0.763	OR = 2.116 1.42–3.154 *p* < 0.001	1.0(ref)	OR = 2.467 2.063–3.194 *p* < 0.001	OR = 1.265 0.963–1.66 *p* = 0.091	OR = 2.506 1.654–3.796 *p* < 0.001

Model 1: Crude model

Model 2: Adjusted for age, gender, smoking and alcohol

**Fig. 3:**
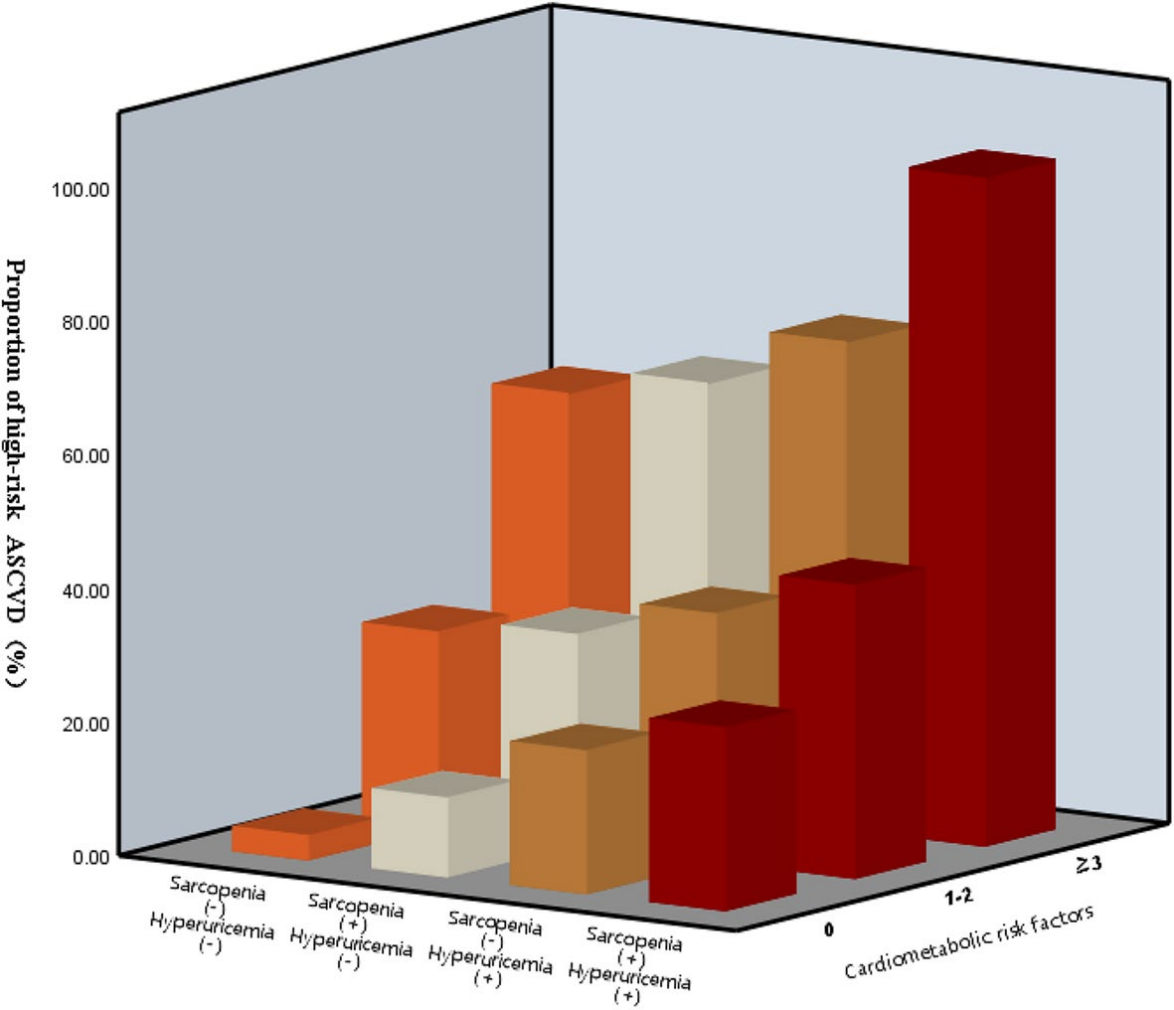
Cardiometabolic risk factors and proportion of high-risk ASCVD

Furthermore, without taking into account the combination of sarcopenia and hyperuricemia, the more cardiometabolic risk factors they combine, the greater the likelihood of high-risk ASCVD occurring when these two conditions are considered separately (Fig. 3). The Y- axis of the Fig. 3 represents the grouping of sarcopenia and hyperuricemia. The Y- axis represents the number of cardiovascular risk factors. The Z-axis represents the proportion of high-risk ASCVD.Cardiometabolic risk factors include dyslipidemia, hypertension, type 2 diabetes, chronic kidney disease, and obesity.

### Association between high-risk ASCVD and hyperuricemia/ sarcopenia

We adjusted for sex, age, smoking, and alcohol consumption in the regression model (Table 3) to further clarify whether hyperuricemia and sarcopenia were independent risk factors for high-risk ASCVD. The results of the regression model analysis revealed that hyperuricemia was an independent risk factor for high-risk ASCVD (OR = 1.355, 95% CI = 1.000–1.838, *p* = 0.04); but the sarcopenia was not(OR = 1.274,95%CI = 0.828–1.959,*p* = 0.271). In addition, the analysis of the association between skeletal muscle mass and uric acid levels is provided in Supplementary material. Our study found a positive correlation between uric acid levels and skeletal muscle mass(β = 0.354,95%CI = 0.016–0.02,*p* < 0.001). After adjusting for confounders, the two remain relevant (β = 0.1, 95%CI = 0.004–0.007, *p* < 0.001) ( Supplementary Table 1).

**Table 3:** ORs and 95% CIs for high-risk ASCVD contributed by sarcopenia and hyperuricemia

	Model 1	Model 2	Model 3
Hyperuricemia	OR = 1.743 95%CI 1.439–2.11 *p* < 0.001	OR = 1.393 95%CI 1.093–1.776 *p* = 0.007	OR = 1.355 95%CI1.000–1.838 *p* = 0.04
Sarcopenia	OR = 1.593 95%CI 1.231–2.062 *p* < 0.001	OR = 1.935 95%CI 1.364–2.745 *p* < 0.001	OR = 1.274 95%CI 0.828–1.959 *p* = 0.271

Model 1: Crude model

Model 1: Adjusted for gender, age, smoking, alcohol

Model 3: Model 2 and diabetes, hypertension, hyperuricemia CKD, hyper-LDL cholesterolaemia, and hypo-HDL cholesterolaemia, diabetes medication, statins, hypertension medication, obesity

**Fig. 4:**
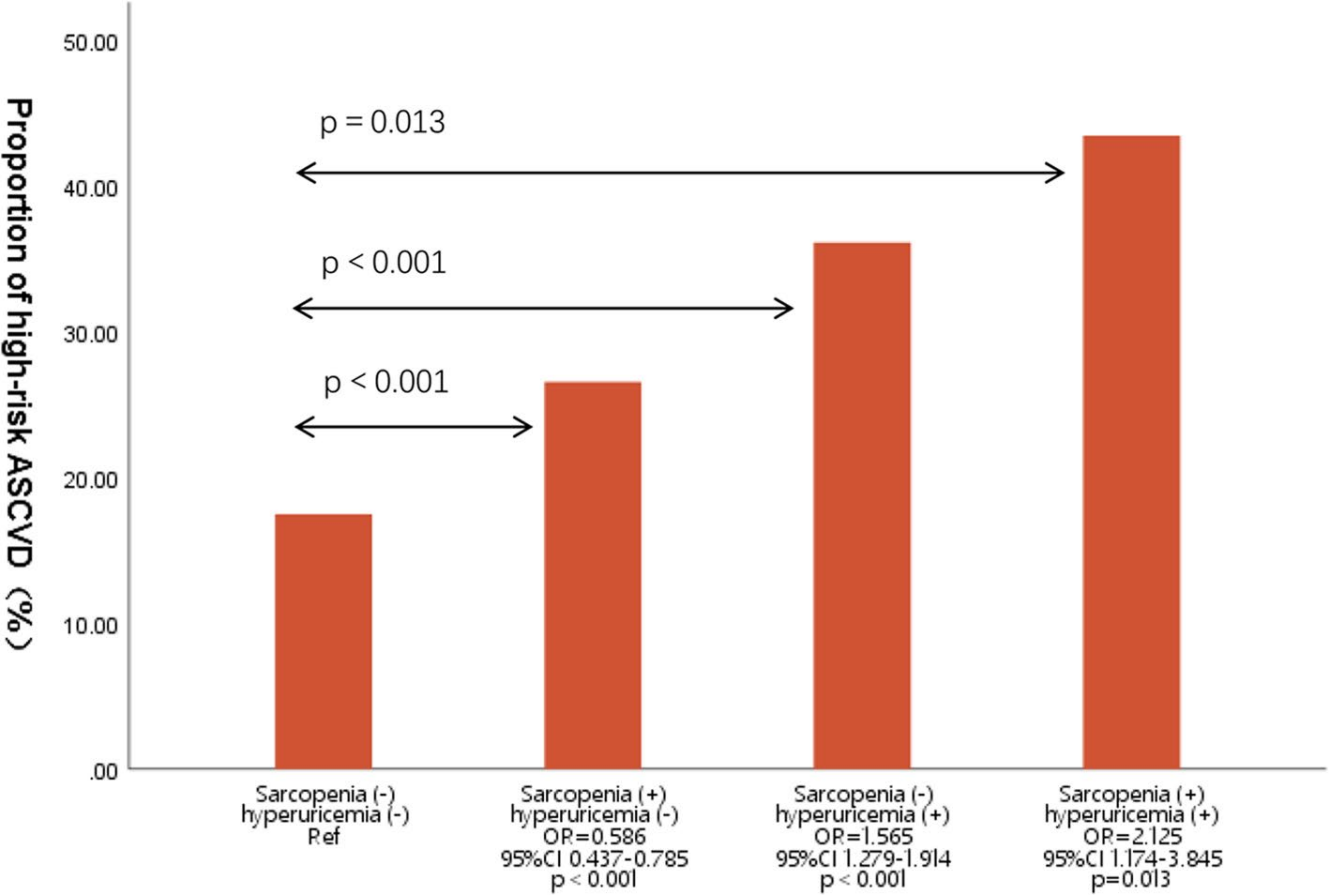
Proportion of high-risk ASCVD by group

According to the status of sarcopenia and hyperuricemia, we further assessed the relative risk of ASCVD (Fig. 4). Without taking sarcopenia into account, subjects with hyperuricemia had a significantly higher prevalence of high-risk ASCVD compared to subjects without hyperuricemia (36.15% vs 17.15%; OR = 1.565, 95%CI = 1.279–1.914); Considering only subjects with hyperuricemia, the prevalence of high-risk ASCVD was 43.47% vs 26.57% in the sarcopenic group(OR = 2.125,95%CI = 1.174–3.84) and the non-sarcopenic group; (OR and 95% CI 2.125, 1.174–3.84, 1.565, 1.279–1.914, respectively). Although sarcopenia did not show an association with high-risk ASCVD after adjusting for confounders, the effect of the two superimposed on ASCVD risk was significantly increased when sarcopenia was combined with hyperuricemia(OR = 3.229,95%CI 1.544–6.751,*p* = 0.002) (Table 4).

**Table 4:** OR and 95% CIs of high-risk ASCVD according to the status of sarcopenia and hyperuricemia

Multivariate models	Sarcopenia (-) hyperuricemia (-) *N* = 1610	Sarcopenia ( +) hyperuricemia (-) *N* = 510	Sarcopenia (-) hyperuricemia ( +) *N* = 413	Sarcopenia ( +) hyperuricemia ( +) *N* = 114
Model 1	1(Ref.)	OR = 0.586 95%CI 0.437–0.785 *p* < 0.001	OR = 1.565 95%CI 1.279–1.914 *p* < 0.001	OR = 2.125 95%CI 1.174–3.845 *p* = 0.013
Model 2	1(Ref.)	OR = 0.907 95%CI 0.649–1.267 *p* = 0.566	OR = 1.234 95%CI 0.988–1.541 *p* = 0.064	OR = 2.704 95%CI 1.362–5.367 *p* = 0.004
Model 3	1(Ref.)	OR = 1.639 95%CI 1.099–2.443 *p* = 0.015	OR = 1.041 95%CI 0.788–1.375 *p* = 0.779	OR = 3.229 95%CI 1.544–6.751 *p* = 0.002

Model 1: Adjusted for age and gender

Model 2: Adjusted for age, gender, smoking and alcohol

Model 3: Adjusted for age, gender, smoking, alcohol, hypertension, diabetes, chronic kidney disease, obesity, hyper-LDL cholesterolaemia, and hypo-HDL cholesterolaemia and medications

## Discussion

According to the findings of our investigation, both sarcopenia (OR = 1.593, 95% CI = 1.231–2.062) and hyperuricemia (OR = 1.743, 95% CI = 1.439, 2.11) were linked to high-risk ASCVD.Hyperuricemia and sarcopenia had synergistic effects on ASCVD risk after adjusting for confounding factors.(OR 3.229, 95% CI = 1.544–6.751). Additionally, hyperuricemia patients with sarcopenia had greater prevalences of cardiometabolic risk factors such as hypertension, obesity, and dyslipidemia.

Our study used high-risk ASCVD as the dependent variable, adjusting for factors such as traditional cardiovascular risk factors, and discovered a 1.335-fold increase in high-risk ASCVD in subjects with hyperuricemia. Elevated uric acid levels are closely related to the development of hypertension, diabetes and metabolic syndrome [27–29]. Extensive prospective studies have found an association between hyperuricemia and atherosclerotic cardiovascular disease [30, 31]. Hyperuricemia is a metabolic disease that occurs earlier than other cardiometabolic diseases such as hypertension and diabetes mellitus [32]. The Mendelian randomized study found that elevated uric acid levels significantly increased the risk of cardiovascular death (OR = 1.77, 95%CI = 1.12–2.81) [33]. Increased uric acid levels have been shown in studies to stimulate the proliferation of vascular smooth cells, decrease the activity of vascular nitric oxide, and contribute to the process of insulin resistance, ultimately leading to atherosclerosis and the formation of atheromatous plaques [3]. Chronic low-grade inflammation is well known to impair the function of endothelial cells, smooth muscle cells, and macrophages, all of which are involved in atherosclerosis, the formation of atheromatous plaques, and eventually atherosclerotic cardiovascular disease [34–37]. In our study, neutrophil levels were significantly higher in those with hyperuricemia only compared to those without hyperuricemia.

Sarcopenia is caused by a number of different pathophysiological mechanisms. Skeletal muscle cells secrete various myokines to communicate with other tissues. Myokines regulate energy expenditure, insulin sensitivity, lipolysis, free fatty acid oxidation, adipocyte browning, and glycogen synthesis and catabolism, and sarcopenia may play a role in the progression of cardiovascular disease via these mechanisms [17, 18]. Hypertensive patients have a relatively high incidence of sarcopenia, and among the many antihypertensive drugs, renin angiotensin-converting enzyme inhibitors may slow the progression of sarcopenia by stimulating muscle synthesis [38]. In this study, sarcopenia was found in 29.55% of hypertensive patients. In non-hypertensive people, the proportion of people with sarcopenia was 19.21%. Although sarcopenia and high-risk ASCVD did not show an independent correlation, we combined hyperuricemia and sarcopenia and found a significant synergistic effect of the combination on the development of high-risk ASCVD. This point is worth discussing.

In this study, TG/HDL was significantly increased in subjects with hyperuricemia and/or sarcopenia compared to subjects without hyperuricemia and sarcopenia. The insulin clamp test, which is the gold standard for identifying insulin resistance [39], is difficult to perform both in clinical and primary care settings. Therefore, some studies have proposed the TG/HDL ratio as a simple alternative indicator of insulin resistance [40–42]. Our previous study also showed that TG/HDL ratio could be used as a simple proxy for insulin resistance to indicate a high risk of metabolic syndrome [43]. Insulin resistance is defined as a decreased sensitivity or reactivity to insulin's metabolic actions, including insulin-mediated glucose handling [44]. It has been demonstrated that insulin resistance affects NO release, which has been linked to vascular fibrosis and vascular sclerosis [45]. Its close association with obesity, diabetes, chronic kidney disease and hypertension as well [43]. We know that insulin resistance plays an important role in the development of ASCVD [46–49]. Therefore, we hypothesized that hyperuricemia and/or sarcopenia may contribute to the development of ASCVD by increasing insulin resistance. Further studies are needed to confirm this.

It is also worth discussing the association between uric acid levels and sarcopenia [20, 21, 50]; The majority of pertinent research conducted thus far include racial and population-specific limitations. Some research investigate the relationship between oxidative stress and sarcopenia based on the dual role of uric acid in this process [51, 52]. In addition, chronic inflammation plays a role in the development of ASCVD, and altered levels of inflammation in patients with hyperuricemia and sarcopenia can affect the development of cardiovascular disease [2–5, 13]. Our findings also show that the NRL ratio, a marker of systemic inflammation that is closely linked to cardiovascular disease [53–55], is much greater in individuals with both co-morbidities. This may also explain why the combination of the two diseases has a greater impact on ASCVD, so further studies are needed to confirm the interaction of signaling pathways related to chronic inflammation in vivo on the two diseases.

## Conclusion

Our study has the following strengths and limitations. First off, our study had a sizable sample size (*n*= 2647) and the association study of high-risk ASCVD with sarcopenia and hyperuricemia was statistically reliable. Similar to earlier studies [56, 57],the prevalence of hyperuricemia (23.57%) and sarcopenia (15.34%) was also observed. This could imply that our study population was properly selected and is somewhat representative of reality. Second, to the best of our knowledge, this is the first study to look into the synergistic effect of hyperuricemia and sarcopenia in high-risk ASCVD patients. Of course, we must be aware of the study's limitations. We were unable to assess the longitudinal dynamic association between changes in hyperuricemia status and changes in muscle mass and ASCVD risk due to the study's cross-sectional design. Furthermore, we only used the muscle mass index to classify sarcopenia, leaving out information on muscle strength and function. The limitations mentioned above may skew the final results. As a result, we will conduct a larger multicenter prospective study at a later date.

## Supplementary Information

**Additional file 1:** **Supplementary table 1.** Linear regression analysis between uric acid and skeletal muscle mass. **Supplementary figure 1.** Scatterplot between uric acid and skeletal muscle mass.

## Data Availability

The datasets used and/or analysed during the current study available from the corresponding author on reasonable request.
