# Supplementary material for: Association of hyperuricemia combined with sarcopenia on ASCVD risk
Source: BMC Cardiovasc Disord. 2023 Jun 27;23:325. doi: 10.1186/s12872-023-03336-2 (PMC10294447; doi:10.1186/s12872-023-03336-2)
Supplement: Supplementary file 1 — Additional file 1: Supplementary table 1. Linear regression analysis between uric acid and skeletal muscle mass. Supplementary figure 1. Scatterplot between uric acid and skeletal muscle mass. [file 12872_2023_3336_MOESM1_ESM.docx]

| Supplementary table 1: Linear regression analysis between uric acid and skeletal muscle mass | | | |
| --- | --- | --- | --- |
| Model | β | 95%CI | p value |
| Model 1 | 0.354 | 0.016-0.02 | ＜0.001 |
| Model 2 | 0.1 | 0.004-0.007 | ＜0.001 |
| Model1：Crude model. Confounding factors are not adjusted;  Model 2：Adjusted for “gender, age, smoking, alcohol, diabetes, hypertension, CHD, CKD.” | | | |

Supplementary Figure


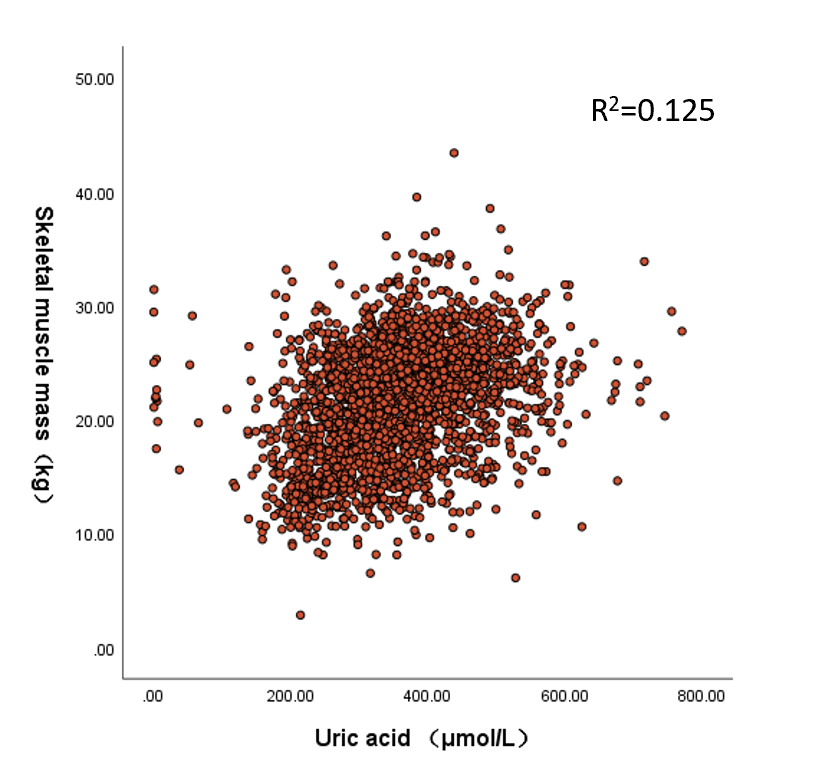


Supplementary figure 1: Scatterplot between uric acid and skeletal muscle mass
